# Radiomic Biomarkers of Locoregional Recurrence: Prognostic Insights from Oral Cavity Squamous Cell Carcinoma preoperative CT scans

**DOI:** 10.21203/rs.3.rs-3857391/v1

**Published:** 2024-01-22

**Authors:** Lei Ren, Xiao Ling, Gregory Alexander, Jason Molitoris, Jinhyuk Choi, Lisa Schumaker, Ranee Mehra, Daria Gaykalova

**Affiliations:** University of Maryland School of Medicine; University of Maryland School of Medicine; Thomas Jefferson University; University of Maryland School of Medicine; Kosin University Gospel Hospital; University of Maryland School of Medicine; University of Maryland School of Medicine; Sidney Kimmel Comprehensive Cancer Center, Johns Hopkins University; Marlene & Stewart Greenebaum Comprehensive Cancer Center, University of Maryland Medical Center; Institute for Genome Sciences, U

## Abstract

This study aimed to identify CT-based imaging biomarkers for locoregional recurrence (LR) in Oral Cavity Squamous Cell Carcinoma (OSCC) patients. Our study involved a retrospective review of 78 patients with OSCC who underwent surgical treatment at a single medical center. An approach involving feature selection and statistical model diagnostics was utilized to identify biomarkers. Two radiomics biomarkers, Large Dependence Emphasis (LDE) of the Gray Level Dependence Matrix (GLDM) and Long Run Emphasis (LRE) of the Gray Level Run Length Matrix (GLRLM) of the 3D Laplacian of Gaussian (LoG σ = 3), have demonstrated the capability to preoperatively distinguish patients with and without LR, exhibiting exceptional testing specificity (1.00) and sensitivity (0.82). The group with LRE > 2.99 showed a 3-year recurrence-free survival rate of 0.81, in contrast to 0.49 for the group with LRE ≤ 2.99. Similarly, the group with LDE > 120 showed a rate of 0.82, compared to 0.49 for the group with LDE ≤ 120. These biomarkers broaden our understanding of using radiomics to predict OSCC progression, enabling personalized treatment plans to enhance patient survival.

## Introduction

Oral Cavity Squamous Cell Carcinoma (OSCC) is the most common malignancy in the head and neck region and is characterized by a poor prognosis [1]. Surgery is the primary treatment of OSCC, followed by cisplatin-based chemotherapy and/or radiotherapy depending on pathologic features and individualized risk of recurrence. Regional recurrence is the most common cause of failure after treatment of oral carcinoma [2, 3]. Despite advancements in surgical techniques and adjuvant therapies, the 5-year overall survival rate hovers between 45–50%, contingent upon the stage and metastasis status of the disease [4, 5]. Locoregional recurrence (LR), as indicated by prior studies [6, 7], represents a significant clinical challenge, with some patients cohorts demonstrating extremely high rates of LR even following surgery and appropriate adjuvant therapy [8]. Given that disease recurrence is devasting for patients, and adjuvant therapies are associated with significant economic and quality of life detriment, identification of patients at higher risk of LR who would benefit most from adjuvant treatment is paramount.

It is, therefore, relevant to identify patients who are at a higher risk of locoregional recurrence before their primary surgery to guide treatment plans and increase the therapeutic window. By utilizing noninvasive imaging information and cutting-edge machine learning algorithms, post-treatment failure can be better screened, enabling medical professionals to tailor treatment plans accordingly.

Histopathologic factors are used for OSCC diagnosis and prognosis staging evaluation [9]. Studies [10-15] reported that tumor size, depth of invasion (DOI), stromal, vascular, and nerve invasion are significantly different between the groups with and without metastasis. The dysregulation of specific miRNAs in OSCC, such as miRNA-184 [16], miR-31 [17], and miR-27b [18], are implicated in malignant transformation and disease progression. Other proteins and peptides, such as Leukotriene A4 hydrolase (LTA4H) and its peptide, Pep8_LTA4H, among other proteins and peptides, may distinguish individuals with metastasis (N+) from individuals metastasis-free (N0) [19]. Studies [20-22] implicated that the amplification of CCND1 and overexpression of cyclin D1 are significantly correlated with OSCC metastasis. Soluble factors, such as IL-1*β*,TNF*α*, and MIP-1*β*, that can be detected in saliva, may also play a significant role in detecting metastasis [23]. The other study [24] reveals an association between primary site recurrence and a high ratio of ITGA3/CD9. Elevated levels of squamous cell carcinoma antigen (SCC-Ag) in serum are significantly associated with tumor progression. [25]. While these parameters evaluated in preclinical settings hold promise in enhancing disease detection, prognosis, and personalized treatment, those findings need to be confirmed by larger and more rigorous studies. One of the limitations of histopathologic biopsy is that it may not capture the full heterogeneity of the tumor due to sampling bias [26]. Furthermore, factors such as DOI are only available on the resection specimen. The extraction and analysis of biomarkers, such as H&E staining, tissue microarray, and sequencing, can be technically complex and expensive, requiring specialized resources, which may restrict their practicality in specific circumstances. Additionally, challenges with reproducibility and standardization across laboratories and the potential for false positives and negatives further complicate their practicality. [27, 28]. Furthermore, validating a biomolecule-based assay, from its initial discovery to clinical implementation, is often arduous and lengthy. A significant number of potential markers prove to be ineffective across various populations [29]. Additionally, it is crucial to reduce the overall processing time to avoid LR in patients who require adjuvant therapy. Also, while some biomarkers may indicate the presence of a disease, they might not offer actionable information for treatment plans, thereby restricting their practical clinical use [30, 31]. Lastly, employing genetic and other biomolecular markers raises ethical, legal, and societal concerns [32]. This is a primary reason these biomarkers have not been introduced in clinical settings and lack FDA approval.

Imaging-based biomarkers have been investigated for different modalities, such as Computed Tomography (CT), Positron Emission Tomography (PET), and Magnetic Resonance Imaging (MRI), by extracting quantitative imaging features known as radiomics features. In contrast to biomolecule-based assays, imaging techniques are non-invasive. The imaging information is readily available from routine diagnostic scans without incurring additional costs. Moreover, imaging provides unique 3D information about neoplasm. These radiomics features can be leveraged to develop predictive models for survival and treatment failure [33-40]. The rationale behind this approach is that these images capture crucial information about the neoplasm phenotype and microenvironment [41]. In fact, the American College of Radiology has developed a standardized Neck Imaging Reporting and Data System (NI-RADS) [42] to manage and surveil the posttreatment course. Studies [43, 44] demonstrated a strong association between NI-RADS category and treatment failure in HNSCC patients. Over recent decades, imaging factors have demonstrated their capacity to furnish accurate prognostic information for posttreatment recurrence screening. A study [45] found a significant association between PET/CT radiomic features and Head and Neck locoregional recurrence. Our pilot study [46] demonstrated two potential Radiomic overall survival biomarkers. However, the identification of non-invasive factors for 2-year locoregional recurrence after primary surgery in OSCC patients remains lacking. The distinction between our study and similar research lies in the emphasis on the susceptibility/risk associated with the biomarker, specifically an increased likelihood of developing locoregional recurrence (LR) within 2 years post-surgery. Consequently, we focus more on specificity and sensitivity to minimize the incidence of false positives and false negatives.

This study aimed to identify CT-based imaging risk factors for locoregional recurrence in patients with OSCC at an academic health network serving a diverse population, which enabled the development of machine learning classifiers that could accurately distinguish patients with locoregional recurrence from those without prior to treatment. A retrospective study design was used, with high-dimensional radiomics, pathological, and clinical information collected from this diverse cohort of OSCC cases. The primary endpoint was 2-year locoregional recurrence (defined as locoregional recurrence occurring within 2 years of surgery). The findings of this study lay the foundation for the implementation of pre-treatment screening for LR and risk assessment using non-invasive risk factors in this diverse patient population, which could ultimately impact the management of high-risk OSCC patients by helping physicians customize treatment planning and reduce the chance of distant metastasis.

## Results

### Oral cavity squamous cell carcinoma cohort features.

This retrospective biomarker analysis examines a group of oral cavity squamous cell carcinoma (OSCC) patients who underwent surgical/curative/elective neck/selective neck resection at the institution between 2006 and 2017. The study involved 78 patients, with 21 experiencing locoregional recurrence (LR), while 57 remained disease-free within a 2-year period after the end of the initial treatment course. Demographic and clinicopathological features of patients are detailed in Table 1. The mean age at the initial surgery was 60, ranging from 30 to 98 years. The median follow-up time for recurrence-free survival (RFS) was 56.2 months. A locoregional recurrence was defined as a positive biopsy in the primary site or the cervical lymphatic region after treatment. We collected six clinical characteristics of interest, including age, gender, tobacco usage, alcohol consumption, T-stage, N-stage, and race. All patients were in the first 2 years of follow-up after surgery. Patients were categorized into four T stages (1, 2, 3, and 4) based on the size and extent of the primary tumor. Smoking and alcohol status were self-reported and coded as 1 for Yes and 2 for No. The missing values for smoking and alcohol status were hard coded as 3 due to their substantial representation within the dataset. Smoking status revealed that 60% of the total cohort were smokers, with this figure rising to 72% in the LR subgroup. For alcohol consumption, 40% of the total group reported alcohol use, compared to 48% in the LR subgroup.

All patients underwent surgery treatment as primary treatment, along with chemoradiotherapy (CRT) or radiotherapy (RT). The endpoint in this study was 2-year LR status, defined as whether an LR happened within 2 years after curative treatment. Here, the proportion increased in the LR subgroup (17% in the total group, 29% in LR), indicating a higher prevalence of this intermediate stage in the LR subgroup.

T3 tumors constituted 17% of the cases. The proportion of T3 tumors increases to 29% in the LR subgroup. T4 tumors, which represent the most advanced stage of tumor size and extent, accounted for 23% of the total cohort. The representation of T4 tumors is notably higher in the LR subgroup, constituting 33%. In total, T3 and T4 stages comprise only 31% of the entire group, in contrast to 62% in the LR subgroup.

### Radiomic factors selection and validation.

A preliminary feature selection algorithm identified 8 radiomic factors of discriminative power in LR depicted in Figure 2a. To ascertain the independence of these two radiomic factors from clinical factors and their potential as clinical alternatives, we investigated their interaction with clinical factors, smoking, alcohol I(ETOH), N stage, and T stage. Logistic regression modeling then incorporated the radiomic features with demographic and clinicopathological characteristics to define the final radiomic risk factors. In this comparative analysis of five logistic regression models in Table 2, denoted as Models 1 through 5, we have assessed their performance based on a range of statistical metrics. The Akaike Information Criterion (AIC) is employed as a model selection criterion. At the same time, accuracy (ACC), area under the receiver operating characteristic curve (AUC), sensitivities (Sens), and specificities (Spec) are utilized to evaluate the models' predictive capabilities. It is evident that the 2nd model (clinical-only) exhibits the highest AIC of 80, suggesting a worse fit to the training data (80%) compared to the other models. When considering the training measurements, the model demonstrates the lowest accuracy (0.75) and AUC values (0.46). In regard to the testing measurements on the held-out 20% data, the 2nd model shows the lowest accuracy (0.6) and AUC (0.67) among all models, showcasing its deficiency in generalization. Moreover, the model consistently maintains an unbalanced sensitivity (0.45) and specificity (1), highlighting its inability to make accurate predictions while minimizing false positives and false negatives. These findings collectively underscore the suboptimal performance of the 2nd model and establish it as the least favorable choice when contrasted with the other models in this analysis.

Numerous studies have identified smoking and drinking as risk factors for OSCC patients. Further analysis indicated that including clinical factors didn’t significantly enhance the model's explanatory power (based on deviance analysis in Table 3 via chi-square test, P=0.43). Table 3 presents the Analysis of Deviance results for eight pairs of nested model comparisons, testing the null hypothesis that additional factors have no effects on outcome. Our analysis yielded robust evidence (via χ^2^-test, p-values < 0.0001) supporting the significance of adding radiomic factors in each pair. Augmenting radiomics to include Smoke, ETOH, and T (the first pair) decreased the deviance by 27.68, indicating a significantly better fit of the larger model to the data. The larger model's AUC showed a noteworthy improvement over the smaller model, as depicted in Figure 3a.

### Risk Stratification and Prognostic ability.

Patients were stratified into high- and low-end groups for recurrence-free survival based on the median value of two factors (Fig. 3b). The Kaplan-Meier curves provide compelling evidence of a significant difference in RFS between the high- and low-end groups (Log-rank p < 0.05). Furthermore, the AUCs of logistic regression, incorporating radiomic factors (0.93), corroborate the significant enhancement in discriminative power when compared to clinical factors-only models (Fig. 3a). Nomograms were constructed with radiomic and clinical factors respectively in Fig. 3c. Notably, the addition of clinical features in the full model demonstrates minimal influence on the predicted RFS probability when compared to the radiomic feature-only model.

### Radiomic Features Uncover Hidden Textural Patterns.

Tumor heterogeneity is widely acknowledged as a significant factor associated with tumor progression. The quantification of tumor heterogeneity has assumed a pivotal role in pathological assessments. Radiomic texture analysis presents distinct advantages, such as non-invasiveness and cost-effectiveness, compared to conventional pathological evaluations. Multiple studies [47-50] have underscored the prognostic potential of GLDM (Gray Level Dependence Matrix) and GLRLM (Gray Level Run Length Matrix) features in the evaluation of tumor progression. These features, GLDM and GLRLM, quantify the degree of local variation within an image [51]. LRE (Long Run Emphasis) serves as a metric for assessing the distribution of long run lengths, with higher values indicative of longer run lengths and coarser structural textures. Conversely, LDE (Long Dependence Emphasis) quantifies the distribution of large dependencies, with elevated values denoting larger dependencies and more homogeneous textures. In light of the findings presented in Table 4, it is noteworthy that, when maintaining LDE at a constant value, each unit increment in LRE corresponds to a 79% (1-0.21) decrease in the odds of recurrence as opposed to non-recurrence. Conversely, when keeping LRE at a fixed value, every unit increasing in LDE results in a 5.8% increase in the odds of recurrence. It is essential to recognize that the estimate for the intercept represents the log odds of a patient with hypothetical zero values for LRE and LDE experiencing recurrence, which is calculated to be 0.095. Predicted Probabilities of locoregional recurrence can be found in **supplementaryFigure 4**. This observation underscores a robust association between these two radiomic factors and the risk of locoregional recurrence.

Figure 4 compares two sets of images processed using the Laplacian of Gaussian (LoG) filter. The first three rows depict LoG-filtered results on an Oral Squamous Cell Carcinoma (OSCC) occurring in the tongue area, with varying sigma (σ) values. The last three rows display results for a non-OSCC area of the tongue. Lower sigma values highlight finer structures, while higher sigma values accentuate larger clusters in the tissue [52]. We observed a pattern in the OSCC images: with increasing sigma, there is a reduction in highlights (white regions) in the filtered images, contrary to the non-OSCC images, which maintain a consistent level of highlights. This suggests that squamous cells in OSCC may be more homogeneous than normal cells. Furthermore, we observed circular artifacts in the OSCC images when filtered with larger sigma (σ > 0.4mm), whereas the normal set presents relatively random structures. to normal tissues. These findings support a fundamental histopathological principle: tumor tissues typically exhibit a more anaplastic and infiltrative pattern than normal tissues [53]. This occurs because tumor cells grow unregulated and clonally, leading to the loss of normal differentiation and organization characteristic of healthy tissues.

## Discussion

Several studies [54-56] have demonstrated the statistical significance of the discriminating ability of radiomic features. The Laplacian of an image highlights regions of rapid intensity change [57]. LDE and LRE measure the distribution of low gray-level values, with a higher value indicating a greater concentration of low gray-level values in the tumor CT scan. SRLGLE measures the joint distribution of shorter run lengths with lower gray-level values despite the universal adoption of CT modality in OSCC diagnosis, automatic imaging prognostic evaluation is lacking and subjective. We present a fully automated prognostic evaluation tool to preoperatively detect locoregional failure in oral cavity cancer. The present study aimed to assess the prognostic capabilities of radiomic features in OSCC locoregional recurrence. Our findings demonstrate that analyzing radiomics from pre-treatment CT scans offers valuable insights into risk factors for locoregional failure and serves as prognostic biomarkers in this patient population. Non-invasive risk factors play a crucial role in personalizing treatment planning, particularly in OSCC, due to the involvement of critical neck surgeries. It is well-known that neck surgeries potentially significantly impact the quality of a patien's life. Thoughtful treatment planning has the potential to mitigate the side effects of unnecessary neck surgery. Key findings of our study include two significant radiomic risk factors: Large Dependence Emphasis (LDE) of the Gray Level Dependence Matrix (GLDM) and Long Run Emphasis (LRE) of the Gray Level Run Length Matrix (GLRLM) of the 3D Laplacian of Gaussian (LoG σ = 3) filtered ROIs.

The AUC showed a stable and approximate value of 0.8 with a sensitivity 0.8 and specificity of 0.8 at the optimal threshold, indicating good prognosis accuracy of the classifier. These results highlight the potential of radiomic features, as a biomarker indicator for treatment failure prognosis. Our study standardized voxel spacing in CT images across patients for precise feature calculation and applied gray-level normalization to enhance feature comparability. These steps are crucial for consistent radiomics analysis. Combining Correlation Analysis, Recursive Feature Selection, and Logistic Regression Best Subset Selection, our feature selection process effectively reduced feature space dimensionality while retaining critical prognostic information. This approach helps mitigate bias, overfitting, and multicollinearity in high-throughput data analysis.

The cost of missing a positive diagnosis (Type 2 error) is often higher than false alarms. On the contrary, since neck dissection significantly decreases the quality of life, reducing the false positive rate (Type 1 error) shall be necessary. In fact, study [58] demonstrate that ROC plots in the context of imbalanced datasets can be deceptive. Therefore, our modeling emphasis was placed on increasing sensitivity and specificity with due consideration to AUC. The threshold for positive event classification plays a pivotal role in predictive accuracy. While a threshold of 0.5 is commonly employed in default, this value is often suboptimal for practical applications in real-world studies, particularly in clinical settings where the distribution of positive cases may have an inherent prevalence, thereby elevating the risks of Type I and Type II errors. Both types of errors are of concern in the study since both overtreatment and undertreatment may lead to escalating healthcare costs and potential harm to patients. To mitigate these risks, we propose adopting a threshold that aligns with the natural prevalence of our cohort, specifically a value of 0.28 for this cohort, for final classification. This calibrated threshold aims to optimize two key metrics: high sensitivity, crucial for minimizing Type II errors and thereby maximizing the identification of LR, and high precision, vital for minimizing Type I errors to reduce false alarms. A number of studies [59, 60] have shown that the chance of an OSCC postoperative locoregional disease being diagnosed positive (Sensitivity) after surgery is only 29%. Our approach demonstrated a noteworthy testing AUC of 0.84, prioritizing both high sensitivity (0.82) and specificity (1). This significantly reduces Type I and Type II errors in post-treatment disease screening tests, effectively minimizing overtreatment and undertreatment.

Our study underscores radiomics' promise in OSCC classification, yet it's crucial to consider its limitations. The small sample size and the classification study's nature might influence our model's radiomics feature stability. For a low-biased, variance classification model with two effects, at least 20 events per training set are advisable, necessitating 27 events for a training set comprising 75% of the sample. This requirement could limit our model's flexibility, potentially impacting the diagnostic capability of the radiomics. Moreover, our analysis only involved radiomics features from CT imaging. Future research should explore features from various imaging techniques, like CT and MRI, to heighten prediction precision. Notably, the observed correlation between certain radiomics features and overall survival hints that these features may mirror the tumors' molecular traits. Upcoming studies should integrate genetic data, such as TP53 [13] mutations and P16 overexpression [28], with radiomics to more comprehensively characterize head and neck squamous cell carcinoma and offer a non-invasive, multimodal approach to OSCC outcome prediction.

In conclusion, our study demonstrated the potential of radiomics as an effective tool to predict treatment response in OSCC patients. Incorporating radiomics analysis into clinical practice could improve decision support and enhance patient stratification, reducing both over-treatment and under-treatment to improve outcomes. Moreover, processing the ROI at the level of small tiles provides an additional non-invasive avenue for assessing the spatial heterogeneity within the tumor. The findings from the study pave the way for future investigations through a larger clinical trial to further evaluate the clinical efficacy of radiomics biomarkers for overall survival prediction for OSCC patients.

## Methods

### Data preparation and overall workflow.

**Figure 1:**
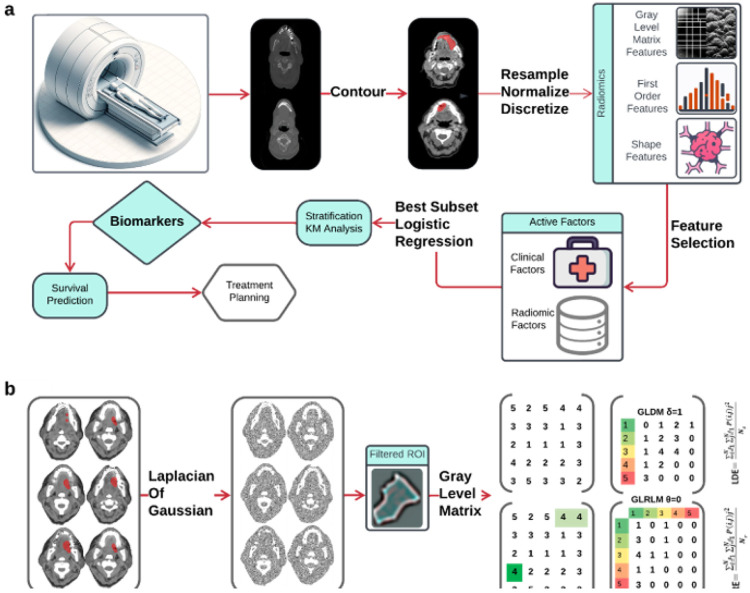
**a** Schematic flowchart illustrating the steps from CT image acquisition and radiomics feature extraction, through the process of machine learning techniques to risk factors. **b** Axial contrast-enhanced CT image of the oral cavity with a red region of interest (ROI) indicating the squamous cell carcinoma. The images were filtered by Laplacian of Gaussian (LoG) and analyzed to extract LDE and LRE features.

The workflow outlining our approach is illustrated in Fig. 1a. In this workflow, the neoplasm volume serves as the region of interest (ROI) from which all radiomics features are computed. The contouring of the ROI was performed manually by experienced Radiation Oncologists, not directly involved in the study, using the Varian Medical System Eclipse software environment. These features underwent a selection process to minimize redundancy and were combined with clinical data. A logistic regression model, optimized via five-repeated 10-fold cross-validation, was then applied. The model's predictive performance was evaluated using the Area Under the ROC Curve (AUC). All statistical analyses were performed using R programming language, with a significance level (alpha) set at 0.05 for all tests.

### Radiomic feature acquisition and extraction.

Uniformity in voxel sizes is essential for precise and dependable feature calculations in radiomics [61]. In our study, the CT scan resolutions varied from 0.3×0.3×0.5 to 1.3×1.3×5.

To standardize, we resampled all CT images to a resolution of 1x1x1mm^3^ using the basis spline algorithm (Bspline) to interpolate the Hounsfield Unit (HU) values in the resampled voxels. Correspondingly, we used the nearest neighbor algorithm to resample the neoplasm contours to the same resolution. Another critical step, gray level normalization, was employed to enhance feature comparability and robustness. As demonstrated in a previous study [61], this normalization minimizes variance and boosts feature robustness of radiomic features, especially against different discretization levels. We normalized the HU values across patients by adjusting the mean and standard deviation, typically resulting in ROI intensities ranging from [−3, 3] after excluding outliers beyond three standard deviations. These intensities were then scaled to a range of approximately [−300, 300]. Moreover, to capture detailed textural information, we discretized the intensities within the ROI using a uniform bin width of 5, starting from a normalized minimum HU value of 0. We chose a bin width of 5 to ensure an adequate number of bins (between 1 and 400), allowing for the capture of more detailed textural information [62]. This discretization assigns new values to each voxel according to the formula: floor((original intensity)/5) + 1. This method not only suppresses noise but also enhances the robustness of radiomic features by smoothing out minor variations.

Medical images provide insights into the phenotypic traits of neoplasms, as depicted in Fig. 4. These images typically contain data from tens of thousands of voxel intensities per neoplasm, leading to a scenario where the number of features (p) greatly exceeds the sample size (n). In our study, we extracted features from each image set using the PyRadiomics library in Python. According to the Imaging Biomarker Standardization Initiative (IBSI) [63], we extracted features across six categories: shape, first-order statistics, gray level co-occurrence matrix (GLCM), gray level run length matrix (GLRLM) [56], grey level size zone matrix (GLSZM) [64], gray level dependence matrix (GLDM) [65], and neighborhood grey tone difference matrix (NGTDM) [66]. Additional calculations were performed on images processed with wavelet, Laplacian of Gaussian (LoG), square, square root, logarithm, exponential, and gradient filters, culminating in 1,092 features.

### Feature selection and modeling.

Logistic Regression Models (LRM) were used in assessing the features of discriminative power. Algorithms are available in the R stats, glmulti package. The guidelines outlined in [67] recommend that the number of predictors used in fitting LRM should not exceed 10% of the events in the sample. In our cohort, with 10% of events in the training sample, the optimal number of predictors for model fitting should be 2 to 3, as 10% of the total recurrence equals 2.1. Thus, we aimed to limit the final Logistic Regression Model (LRM) to a maximum of 3 degrees of freedom. We used the Best Subset Selection (BSS) Modeling strategy to identify the most effective LRM based on validation performance to achieve this. BSS, known for its efficiency in finding the most parsimonious model, outperforms methods like stepwise selection and Lasso, although its high computational demand is a limitation. For instance, fitting LRMs with 2, 3, or 4 degrees of freedom using 17 radiomics and six clinical features requires BSS to estimate a minimum of 41,262 coefficients, making exhaustive evaluation impractical. Therefore, we reduced the number of input variables before employing BSS.

**Figure 2:**
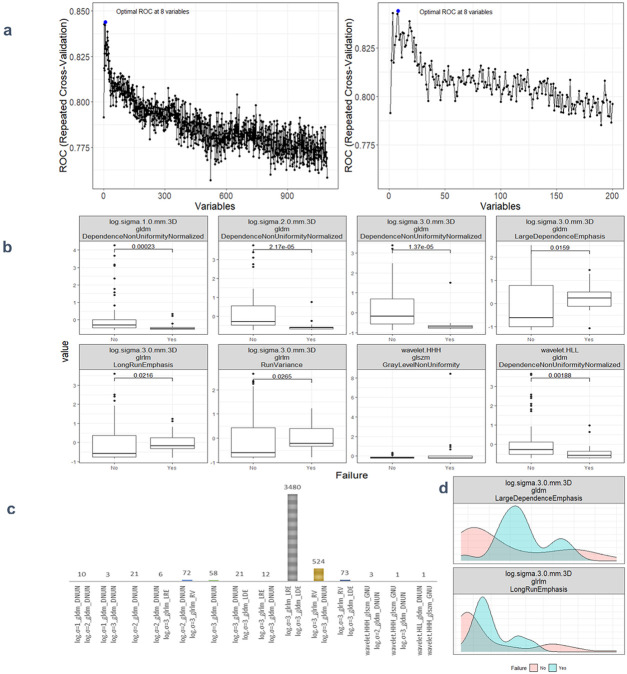
**a** The plot showcases a notable increase in AUC at the retention of eight informative features, followed by a decline in performance as non-informative features are incorporated. Left panel provides a comprehensive view of the AUC achieved across varying numbers of variables. Right panel offers a closer examination of the AUC within the range of 0 to 200 features. **b** This set of box plots presents a comparative analysis of eight distinct radiomics features. The selection process involved repeated 5x10-fold cross-validation RFE. **b** This frequency bar plot visualizes the counts of various degree-2 logistic models derived from the best-subset of eight radiomics features previously identified. These models were generated through 5000 iterations of data shuffling. **d** This density plot illustrates the distribution of two radiomic factors across two groups: recurrence and non-recurrence.

**Figure 3:**
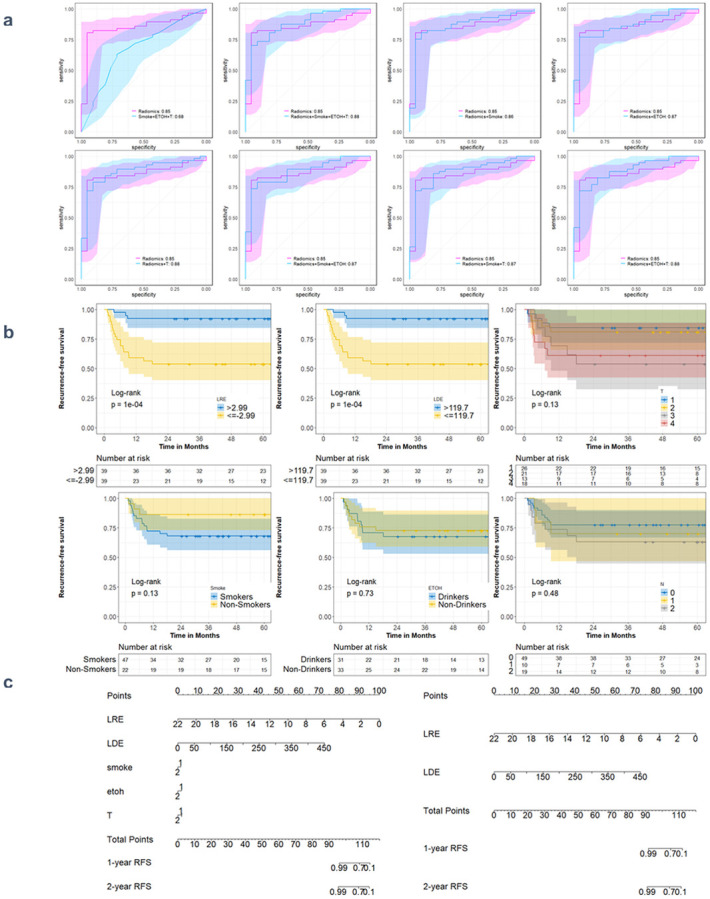
**a** These plots illustrate the logistic ROC, delineating comparisons between the full model, which incorporates previously identified radiomic and clinical features, and alternative combinations. The numbers next to each model in the legend give the AUC. **b** The Kaplan-Meier survival curves demonstrate a significant contrast (p = 1e-04) in recurrence-free survival (RFS) between high/low-end radiomic risk groups. **c** Side-by-side comparison of nomograms illustrating RFS probability predictions for the cohort. On the left, the "Full Model" includes both radiomic and clinical factors, while on the right, the "radiomics-Only Model" consists of two identified radiomic risk factors exclusively.

**Figure 4:**
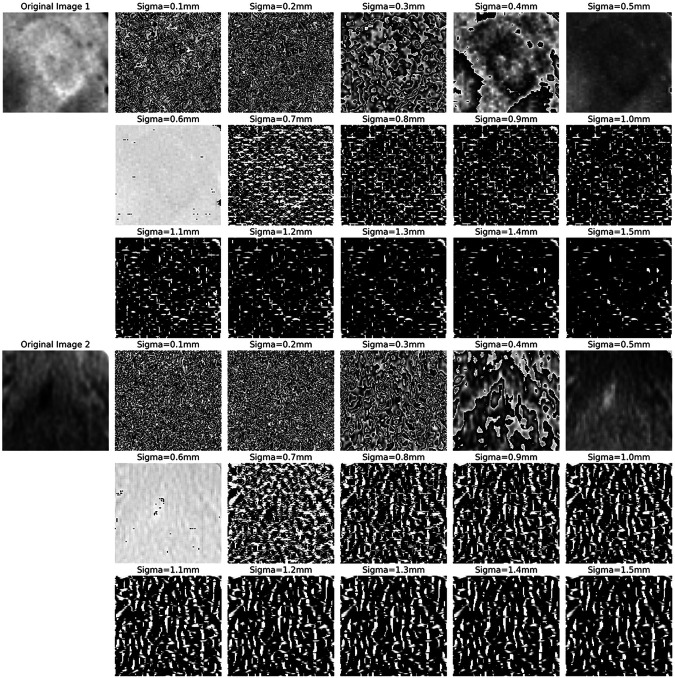
The figure displays two sets of LoG filtered images. The first three rows show OSCC images in the tongue region at varying sigma values, illustrating changes in the primary ROI. The last three rows depict normal tissue in the same region, also filtered at corresponding sigma values, to highlight contrasts between OSCC and normal textures.

**Figure 5:**
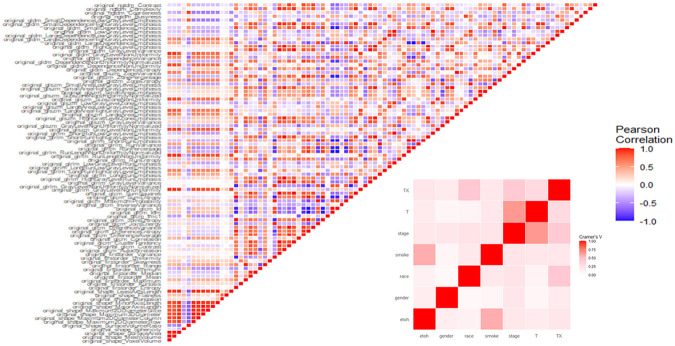
Correlation Coefficient Heatmaps: On the left, the diagonal heatmap illustrates the pairwise correlations among radiomics features before pruning. On the right, the diagonal heatmap demonstrates the correlations of clinical features. The color scale represents the strength of the correlation, with blue indicating negative correlation, red indicating positive correlation, and white representing no correlation.

Radiomics data often faces the challenge of high multicollinearity, where variables are highly correlated, affecting the significance of individual variables in the model. For example, sphericity, minor axis length, and elongation show strong multicollinearity. Multicollinearity can lead to the phenomenon where a variable is not deemed significant when correlated features are also present in the model. Figure 5 uses a color scheme where white represents no correlation, blue represents a perfect negative correlation, and red represents a perfect positive correlation. The heatmap illustrates the correlation coefficients prior to the feature selection process, revealing the initial relationships between features. The heatmap revealed numerous red and blue shades, indicating strong positive and negative correlations, respectively, among the data. There is now a substantial body of research on mitigating multicollinearity, such as Principal Component Analysis (PCA), Sparse PCA [69], and Kernel PCA (KPCA) [70]. We employed Recursive Feature Elimination (RFE), a popular method, to refine the input data for BSS LRM. Using RFE with repeated 10-fold cross-validation (**supplementary Table 1**),, we narrowed down from 1,092 radiomic features to a subset of eight active features (Fig. 2b). We then fitted degree-2 LRMs, considering all combinations of these eight features across 5,000 data shuffles. The models were trained on 63 samples (80% of the cohort) using 10-fold cross-validation and evaluated based on AUC. The remaining 15 samples (20% of the cohort) were used for prognostic validation. We also used the ROC to visualize the classifiers’ performance. Figure 2c shows that the 3,480 models out of 5,000 data shufflings unveiled the most distinctive factor among the extensive array of 1,092 radiomic features: specifically, Large Dependence Emphasis (LDE) of the Gray Level Dependence Matrix (GLDM) and Long Run Emphasis (LRE) of the Gray Level Run Length Matrix (GLRLM) of the 3D Laplacian of Gaussian (LoG _σ=3_) filtered ROIs (Fig. 1b and Fig. 2d). Statistical evidence was quantified by the p-value of the hypotheses’ tests. If the p-value is less than α = 0.05, then the null hypothesis will be rejected in favor of the alternative hypothesis.

### Assessment of the prognosis.

We stratified the corhort into high and low LRE and LDE subgroups according to the threshold selected by using respective median. Kaplan-Meier curve analyses were conducted to assess the impacts of the biomarkers on RFS. RFS was defined as the time from surgery to locoregional recurrence.

### Constructing nomogram.

The primary end-point of the analysis was the time to peritoneal locoregional recurrence. The follow-up duration to peritoneal LR was calculated from the date of surgery to the date when peritoneal LR was diagnosed or to the last follow-up, and information about the survival status and recurrence type was also documented. Finally, a radiomic nomogram was constructed. A model containing both radiomic and clinical factors was also constructed for comparison.

## Figures and Tables

**Table 1:** Frequency and Significance of demographic and clinicopathological characteristics.

	Total (%)	LR (%)
Gender		
Male	45(58%)	14(67%)
Female	33(42%)	7(33%)
Race		
EA	69(88%)	17(81%)
AA	9(12%)	4(19%)
Smoking		
Yes	47(60%)	15(72%)
No	22(28%)	3(14%)
Unknown	9(12%)	3(14%)
Alcohol		
Yes	31(40%)	10(48%)
No	33(42%)	9(43%)
Unknown	14(18%)	2(9%)
T stage		
T1	26(33%)	4(19%)
T2	21(27%)	4(19%)
T3	13(17%)	6(29%)
T4	18(23%)	7(33%)
N stage		
N0	49(63%)	11(52%)
N1	10(13%)	3(14%)
N2	19(24%)	7(34%)
Treatment		
Sx	45(58%)	8(38%)
Sx + RT	18(23%)	5(24%)
Sx + CRT	15(19%)	8(38%)
Registry Sites		
Buccal Mucosa	11(14%)	1(5%)
Floor of Mouth	6(8%)	2(10%)
Gingiva	13(17%)	2(10%)
Retromolar trigone	1(1%)	0(0%)
Tongue	47(60%)	16(75%)

Note: Total, the entire cohort; LR, the locoregional recurrent cohort.

**Table 2:** Logistic Regression Models with Different Factor Inclusions

							Training				Testing			
	Factor	Est.	se	z	*p*- value	AIC	ACC	AUC	Sens	Spec	ACC	AUC	Sens	Spec
1	Intercept	2.646	0.796	3.324	0.001	57	0.79	0.83	0.57	0.87	0.87	0.84	0.82	1.00
	LRE	1.323	0.429	3.086	0.002									
	LDE	−0.051	0.015	−3.486	0.000									
2	Intercept	0.679	0.481	1.411	0.158	80	0.75	0.46	0.23	0.93	0.60	0.67	0.45	1.00
	Non-Smoke	0.160	0.718	0.223	0.823									
	Non-ETOH	0.234	0.666	0.351	0.726									
	T2	−0.856	0.604	−1.418	0.156									
	T3	0.446	0.634	0.704	0.482									
	T4	0.976	0.648	1.505	0.132									
3	Intercept	2.360	0.869	2.715	0.007	58	0.80	0.86	0.57	0.87	0.87	0.86	0.82	0.87
	LRE	1.404	0.465	3.020	0.003									
	LDE	−0.053	0.016	−3.404	0.001									
	Non-Smoke	0.586	0.758	0.773	0.439									
4	Intercept	2.048	0.911	2.248	0.025	58	0.80	0.85	0.53	0.90	0.87	0.89	0.82	0.87
	LRE	1.600	0.532	3.008	0.003									
	LDE	−0.060	0.018	−3.357	0.001									
	Non-Smoke	0.191	0.825	0.232	0.817									
	Non-ETOH	1.125	0.851	1.323	0.186									
5	Intercept	2.076	1.008	2.060	0.039	63	0.76	0.83	0.45	0.87	0.87	0.89	0.82	0.87
	LRE	1.696	0.579	2.931	0.003									
	LDE	−0.063	0.019	−3.308	0.001									
	Non-Smoke	0.247	0.861	0.287	0.774									
	Non-ETOH	1.140	0.888	1.283	0.199									
	T2	0.355	0.765	0.464	0.643									
	T3	−0.161	0.833	−0.193	0.847									
	T4	0.897	0.793	1.131	0.258									

Note: ACC measures overall correctness, AUC assesses discrimination ability, Sen measures the ability of the model to correctly identify positive, Spec measures the ability of the model to correctly identify negative, and AIC indicates how close fitted values to expected values. In this case, the 1st model (AIC=57) is considered more efficient in explaining the observed variation in the data than 2nd model.

**Table 3:** Analysis of Deviance for various Logistic regression Models

Model Comparison	Res.Df.	Res.Dev.	Df.	Dev.	p-value
Radiomics + Smoke + ETOH + T	70	56.74			
Smoke + ETOH + T	72	84.42	−2	−27.68	<0.0001
Radiomics + Smoke	74	60.47			
Smoke	76	89.33	−2	−28.86	<0.0001
Radiomics + ETOH	74	59.34			
ETOH	76	90.13	−2	−30.79	<0.0001
Radiomics + T	72	59.24			
T	74	84.78	−2	−25.53	<0.0001
Radiomics + ETOH + Smoke	73	58.99			
ETOH + Smoke	75	89.10	−2	−30.11	<0.0001
Radiomics + T + Smoke	71	58.60			
T + Smoke	73	84.65	−2	−26.04	<0.0001
Radiomics + T + ETOH	71	56.88			
T + ETOH	73	84.45	−2	−27.57	<0.0001
Radiomics + Smoke + ETOH + T	70	56.74			
Radiomics	75	61.55	−5	−4.81	0.4398

Note: Comparisons assess the impact of radiomics inclusion. The first tests the superiority of the full model (radiomics and clinical) over the clinical model alone, while the rest evaluates the model with or without radiomics. Significant p-values favor the full model in all comparisons. The table details degrees of freedom (Res.Df.), residual deviance (Res.Dev.), changes in degrees of freedom (Df.), changes in deviance (Dev.), and associated p-values.

**Table 4:** Summary of Effects in Model with entire Cohort

Coef.	Estimate	95% CI		OR	95% OR CI		p-value
Intercept	−2.355	−3.759	−0.952	0.095	0.019	0.331	0.001
LRE	−1.557	−2.404	−0.709	0.211	0.076	0.433	0.000
LDE	0.056	0.028	0.084	1.058	1.032	1.093	0.000

Note: SE = standard error, z = z-value, CI = Confidence Interval

## Data Availability

The datasets generated and/or analyzed during the current study are not publicly available due to institutional policy but are available from the corresponding author on reasonable request.

## Code availability

The code used in this study is available at https://doi.org/10.5281/zenodo.10460030.
